# Supplementary material for: Hybrid Models and Biological Model Reduction with PyDSTool
Source: PLoS Comput Biol. 2012 Aug 9;8(8):e1002628. doi: 10.1371/journal.pcbi.1002628 (PMC3415397; doi:10.1371/journal.pcbi.1002628)
Supplement: Text S4 — Complete source code for the PyDSTool package (version 0.88.120504). Includes API documentation and help files linking to web pages. This file is identical to the current public release on Sourceforge.net. (ZIP) [file pcbi.1002628.s004.zip › PyDSTool/html/PyDSTool.Toolbox.phaseplane.phaseplane-class.html]

xml version="1.0" encoding="ascii"?


PyDSTool.Toolbox.phaseplane.phaseplane


| Home | Trees | Indices | Help | PyDSTool |
| --- | --- | --- | --- | --- |

Package PyDSTool ::
Package Toolbox ::
Module phaseplane ::
Class phaseplane

[hide private]

[frames] | no frames]

# Class phaseplane

source code

```
object --+
         |
        phaseplane
```

---

Working environment for 2D phase-plane analysis.

Effectively a thin wrapper around a Generator class (will later be
around a Model class, in order to support hybrid 2D vector fields)


|  |  |
| --- | --- |
| Instance Methods | [hide private] |

|  |  |
| --- | --- |
| \_\_init\_\_(self, vf\_info, x\_axis=`'``'`, name=`'``'`, normord=2, eps=1e-12)  Use x\_axis to specify which variable should appear on the x-axis | source code |

|  |  |
| --- | --- |
| build\_vf(self) | source code |

|  |  |
| --- | --- |
| add(self, attribute, value) | source code |

|  |  |
| --- | --- |
| remove(self, attribute, value) | source code |

**Inherited from `object`**:
`__delattr__`,
`__getattribute__`,
`__hash__`,
`__new__`,
`__reduce__`,
`__reduce_ex__`,
`__repr__`,
`__setattr__`,
`__str__`


|  |  |
| --- | --- |
| Properties | [hide private] |

**Inherited from `object`**:
`__class__`


|  |  |
| --- | --- |
| Method Details | [hide private] |

|  |  |
| --- | --- |
| \_\_init\_\_(self, vf\_info, x\_axis=`'``'`, name=`'``'`, normord=2, eps=1e-12)  *(Constructor)* | source code |

Use x\_axis to specify which variable should appear on the x-axis

Overrides: object.\_\_init\_\_

  


| Home | Trees | Indices | Help | PyDSTool |
| --- | --- | --- | --- | --- |

|  |  |
| --- | --- |
| Generated by Epydoc 3.0.1 on Fri May 4 15:24:10 2012 | http://epydoc.sourceforge.net |
